# University of Louisville

**Published:** 1852-12

**Authors:** 


// THE WESTERN JOURNAL.

Third Series.—Vol. X.—No. VI.

LOUISVILLE, DECEMBER 1, 1852.

UNIVERSITY OF LOUISVILLE.

The number ef Medical students in the University of Louisville is larger, as we go to press to-day, than it was on the corresponding day of last December, so that there is not likely to be any falling off in the class. The number, last session, was 261. Professors Flint and Palmer have fully sustained the reputation which they had acquired at the North as impressive and instructive teachers, and amply justified the choice of the judicious Board of Trustees. If delicacy permitted, we would like to enlarge upon the merits of our colleagues as lecturers, but we feel that we may safely leave their reputation with their pupils.

During the winter, it is the intention of the Faculty to issue a Catalogue of the students from the commencement of the institution, copies of which will be forwarded to the alumni or others desirous of possessing it, on application to the Dean.

We are pleased to learn that our friends in the Law Department have an unusually large class. Talents, attainments, and efforts to build up a first-rate school, such as theirs, ought to be successful, and it may safely be predicted will be.

